# Accurate Size and Size-Distribution Determination of Polystyrene Latex Nanoparticles in Aqueous Medium Using Dynamic Light Scattering and Asymmetrical Flow Field Flow Fractionation with Multi-Angle Light Scattering

**DOI:** 10.3390/nano2010015

**Published:** 2012-01-05

**Authors:** Haruhisa Kato, Ayako Nakamura, Kayori Takahashi, Shinichi Kinugasa

**Affiliations:** 1 Polymer Standards Section Japan (PSSJ), Particle Measurement Section (PMS), National Metrology Institute of Japan (NMIJ), National Institute of Advanced Industrial Science and Technology (AIST), Tsukuba Central 5, 1-1-1 Higashi, Tsukuba, Ibaraki 305-8565, Japan; Email: H-kato@aist.go.jp (H.K); Email: nakamura@tasc-nt.or.jp (A.N); Email: kayori.takahashi@ni.aist.go.jp (K.T); Email: s.kinugasa@aist.go.jp (S.K)

**Keywords:** asymmetric flow field flow fractionation, multi-angle light scattering, dynamic light scattering, nanoparticle, polystyrene latex

## Abstract

Accurate determination of the intensity-average diameter of polystyrene latex (PS-latex) by dynamic light scattering (DLS) was carried out through extrapolation of both the concentration of PS-latex and the observed scattering angle. Intensity-average diameter and size distribution were reliably determined by asymmetric flow field flow fractionation (AFFFF) using multi-angle light scattering (MALS) with consideration of band broadening in AFFFF separation. The intensity-average diameter determined by DLS and AFFFF-MALS agreed well within the estimated uncertainties, although the size distribution of PS-latex determined by DLS was less reliable in comparison with that determined by AFFFF-MALS.

## 1. Introduction

Recently, there has been an unprecedented increase in the number of studies related to nanomaterials [1,2,3,4,5]. Accurate determination of nanomaterial size is crucial for developing nanoscale technologies, because size governs many of the physical and chemical properties of these materials. For example, a good photocatalyst needs a large catalytic surface area and the primary size of catalyst nanoparticles defines the surface area available for adsorption and decomposition of organic pollutants. With regards to the adsorption and reaction of sulfur dioxide on photocatalytic titanium dioxide nanoparticles, the inherent adsorption capacity of the smaller nanoparticles has been found to be larger because of the greater saturated surface coverage of sulfite adsorbed on the nanoparticles [6].

The adverse effects of nanomaterials on human health are a serious issue that has also attracted much attention. Many international organizations and researchers have therefore carried out toxicity assessments of various nanoparticles, such as metals, metal oxides, fullerenes, and carbon nanotubes [7,8,9,10]. The European Commission has declared that a “nanomaterial” is a natural, incidental or manufactured material containing particles, in an unbound state or as an aggregate or as an agglomerate and where, for 50% or more of the particles in the number size distribution, one or more external dimensions is in the size range 1–100 nm [11]. According to this definition, not only the size but also the size distribution of nanomaterials in a liquid phase is an important factor for nanotoxicity assessment. Primary nanoparticle size is commonly determined by the Brunauer, Emmett, and Teller (BET) method or by a microscopic technique such as transmission electron microscopy (TEM). However, nanoparticles sometimes aggregate or agglomerate in a liquid phase, resulting in the formation of secondary particles. In such a case, the sizes obtained by BET and TEM analysis do not reflect the actual secondary particle size and size distribution of nanomaterials in a liquid phase. Furthermore, in TEM analysis, a long time is required to count a sufficient number of nanoparticles for accurate determination of size and size distribution.

Dynamic light scattering (DLS) is widely used as an effective technique for determining the average secondary particle size of Brownian nanoparticles in colloidal suspensions [12]. In that method, the diffusion coefficients of nanoparticles are first determined, and the average diameters of the particles are then calculated from these coefficients by using the Stokes–Einstein relationship. In contrast to the reliable intensity-average hydrodynamic diameter determined by DLS, determination of nanoparticle size distribution in a liquid phase using DLS is not reliable because the method provides only a qualitative analysis of the size distribution from the observed photon correlation function [13]. Indeed, the size distribution of nanoparticles analyzed by DLS strongly depends on the instrument and analytical procedure used [12]. Surprisingly, the problem regarding the poor determination of particle size distribution by DLS is not known to a broad community.

Asymmetric flow field flow fractionation (AFFFF) is an elution technique whereby nanoparticles and macromolecules are separated by flow control in an aqueous medium [14]. In AFFFF, the retention time (*t_r_*) of nanoparticles can be predicted by the Giddings equation:


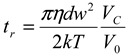
(1)

where *kT* is the thermal energy, *η* is the viscosity of the carrier liquid, *d* is the nanoparticle diameter, *w* is the channel thickness, *V_0_* is the volumetric flow rate through the channel, and *V_C_* is the cross flow rate. Thus, when the cross flow and channel flow rates are constant in the AFFFF system, the separation of nanoparticles by size can achieved and the retention time is proportional to the size of the separated nanoparticles. A drawback to AFFFF separation is band broadening, which results in poor size separation. Band broadening is caused by the different displacement velocities of particles in the flow profile of the AFFFF system. The broadening of particle size bands should therefore be kept to a minimum. One solution to the broadening problem is confinement of particle distribution to a layer whose streamlines have a small velocity range [14]. Such confinement can be accomplished by employing appropriate sample focusing techniques after injection. However, complete elimination of band broadening by controlling the experimental conditions is impossible. Therefore, the effect of band broadening on the apparent size values determined by AFFFF-multi-angle light scattering (MALS) should be estimated by using particles with a known narrow size distribution.

The purpose of this study is to establish a robust protocol for determining the size and size distribution of nanoparticles in a liquid phase by DLS and AFFFF. First, the intensity-average size of polystyrene latex (PS-latex) nanoparticles in an aqueous suspension was accurately determined by DLS using extrapolation of both the PS-latex concentration and the observed scattering angle [15,16,17]. Second, an AFFFF system equipped with a MALS detector was used to determine the mean size and size distribution of PS-latex nanoparticles. To determine a reliable size distribution of PS-latex nanoparticles, the effect of band broadening on the apparent size distribution determined by AFFFF-MALS was examined. The size distribution determined in the gas phase by differential mobility analysis (DMA), which is commonly used to determine the size distribution of nanoparticles [18], was employed to examine the effects of band broadening on the apparent nanoparticle size distribution obtained by AFFFF-MALS. Then, we estimated an accurate size distribution of PS-latex after taking into account the effect of band broadening on the apparent values. Although the importance of considering band broadening in the quantitative determination of the nanoparticle size distribution by AFFFF-MALS is well known, the quantitative analysis in this study is novel in FFFF research. Third, the carefully determined average size of PS-latex by DLS was compared with that by AFFFF-MALS. To compare the two values obtained by DLS and AFFFF-MALS, we examined the sources and magnitudes of errors in the two methods. In this study, the Guide to the Expression of Uncertainty in Measurement (GUM) was employed, which is published by the Joint Committee for Guides in Metrology. This committee includes international standards organizations such as the Bureau International des Poids et Mesures (BIPM) and the International Organization for Standardization (ISO). Using this precise assessment of the uncertainties for both DLS and AFFFF-MALS results, we were able to compare the values determined by the two methods, quantitatively. We believe that the establishment of a practical protocol for the determination of the size and size distribution of particles by DLS and AFFFF-MALS will be of great benefit not only in nanotechnology and biology, but also in wide range of other disciplines such as chemistry, physics, technology, toxicology and others. A well-defined protocol is needed to provide a basis for reliably comparing different results for functional nanomaterials, with respect to the size and size distribution of particles in suspension. Furthermore, the reliability of DLS and AFFFF-MALS for determining size distribution was investigated. We focused on PS-latex (T0625, see Table 1) and carefully characterized it.

**Table 1:** Characteristics of PS-latex nano particles used in this study.

Sample name	Official diameter ^a)^	CV value ^b)^
(nm)	%
STADEX SC-0070-D	70	7.30
STADEX SC-0080-D	80	4.80
STADEX SC-0100-D	100	2.47
STADEX SC-0110-D	107	3.10
STADEX SC-0140-D	144	1.42
STADEX SC-016-S	152	2.46
F0223	140	-
T2112	147	-
T0622	128	-
T0021	90	-
T0118	91	-
T0408	88	-
T0625	103	-
^a)^ The official values of official diameter are determined by DMA or TEM; ^b)^ CV values are calculated from the standard deviation of the size distribution by DMA, and the observed size distributions are divided by size.

## 2. Experimental Section

### 2.1. Materials

Surfactant-free aqueous suspensions of approximately 10 wt % PS-latex nanoparticles were used (T0625, JSR Co., Tokyo, Japan). The suspension was diluted with ultrapure water from a Milli-Q system (Nihon Millipore K.K., Tokyo, Japan) using 0.1 μm filters. The characteristics of the suspension are summarized in Table 1.

### 2.2. DLS Measurements

The dynamic light scattering apparatus (DLS7000, Otsuka Electronics Co., Ltd., Kyoto, Japan) used in this study has goniometer equipped with a 35 mW He-Ne laser of 632.8 nm wavelength. A multiple tau digital correlation scheme was used with a minimum sampling time of 0.1 μs. The measurements were performed at a scattering angle of 90°. A quartz sample cell was set in a silicon oil bath such that the refractive indices of the oil and the cell were nearly equal. Light scattering was measured at a regulated temperature of 25.0 ± 0.1 °C. Values reported in this paper are expressed as the mean of three replicates.

Assuming a dilute suspension of monodisperse particles (*i.e.*, all the particles are of the same size and shape) and no interaction between particles, we have


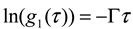
(2)

Here, 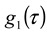 is the homodyne correlation function in DLS and Γ is the decay rate related to the translational diffusion coefficient (D) of the particles in the medium by 


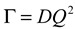
(3)

where the modulus of the scattering vector is given by


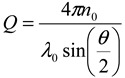
(4)

Here, *θ* is the observed angle, *λ_0_* is the wavelength of the laser source in vacuum, and *n_0_* is the refractive index of the solvent medium. 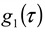 thus obtained was analyzed by the first cumulant method described in ISO 13321 to determine the average decay rate Γ [19]. The apparent diffusion coefficient obtained from DLS was used in the Stokes–Einstein relation of the following form to determine the hydrodynamic particle size of the PS-latex secondary nanoparticles:


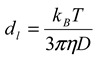
(5)

Here, *k_B_* is the Boltzmann constant, *T* is the absolute temperature, *η * is the viscosity of the solvent (0.8902 cP) [20], and *d *is the calculated hydrodynamic diameter of the nanoparticles.

### 2.3. AFFFF-MALS Measurement

Size determination by AFFFF-MALS was carried out on a system (AF2000 FFF, Postnova, Germany) equipped with a cellulose membrane (Z-MEM-AQU-427N) with a molecular weight cutoff of 10,000 Da and a channel thickness of 350 μm. The main carrier flow and the focusing flow were provided by two double pumps (PN1122, Postnova, Germany). The carrier water was degassed on-line by a vacuum degasser (PN7505, Postnova, Germany). The constant cross flow rate was changed from 0.18 mL/min to 0.25 mL/min, and the channel flow was maintained at a constant 1.0 mL/min. To determine the size of the nanoparticles in each fraction separated by AFFFF, a MALS detector (Dawn EOS, Wyatt Technology Co., USA) was used. Light scattering was detected at 690 nm. The MALS detectors were calibrated using pure toluene, and the detectors at different angles were normalized with respect to a 90° detector measuring bovine serum albumin monomer separated by AFFFF. After AFFFF separation, the nanoparticle sizes in the fractions were determined by the MALS detectors over 11 scattering angular ranges from 34.8° to 152.5°. The intensity distribution function is given by


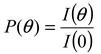
(6)

Assuming spherical particles (the diameter is *d_s_*) to be measured, the intensity distribution function is given by


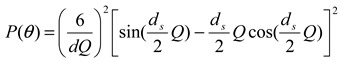
(7)

Since the experimental results agree well with calculation results from Equation 7, the assumption of spherical PS-latex nanoparticles is valid. The size of particles was calculated by the ASTRA software particle module analytical method.

Ultrapure water (18.2 MΩ cm electrical resistance, and

## 3. Results and Discussion

### 3.1. DLS

#### 3.1.1. Calculation Method for Reliable Size Determination by DLS

According to the Stokes–Einstein relation (Equation 5), the intensity-average hydrodynamic diameter *d_l_* of the particles can be calculated from the diffusion coefficient (*D*) of particles. In principle, the particle concentration in suspension and the optical path length must be sufficiently low to prevent multiple scattering. For a higher concentration suspension, a scattered photon has non-negligible probability of being re-scattered when passing through the suspension. The autocorrelation function for such multiple scattering decays faster than the one for single scattering, resulting in underestimation of particle size. However, if the particle concentration is too low, the scattering is unstable. DLS measurements can also be easily affected by traces of dust. A solution to this problem is to extrapolate the apparent diffusion coefficients observed at a large number of concentrations to infinite dilution. Doing so yields more reliable results than those obtained for measurements at only one concentration. In this study, the measured apparent intensity-average hydrodynamic diameter was therefore extrapolated to infinite dilution and to zero angle in order to determine the true hydrodynamic size in an aqueous medium. We refer to the double extrapolation plot as a Zimm plot in this paper.

#### 3.1.2. Size Determination of PS-Latex by DLS

**Figure 1:**
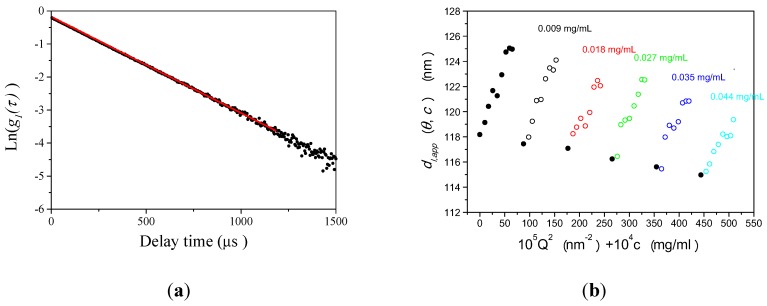
**a**) Example of observed homodyne photon correlation function for PS-latex (T0625) analyzed by dynamic light scattering ( DLS) (filled circle plots). The concentration of the aqueous PS-latex suspension was 0.009 mg/mL and light scattering was observed at a scattering angle of 90 ° . The red line is the linear least-squares fit of the experimental results. (**b**) Example of the constructed Zimm plots for PS-latex (T0625) analyzed by DLS at a concentration range from 0.009 to 0.044 mg/mL. Light scattering was observed at 8 different scattering angle s from 45° to 150° . The true *d_l_* was calculated to be 118.2 nm.

Figure 1a shows an example of the plot of the raw photon correlation function of PS-latex determined by DLS measurement. The concentration of the aqueous suspension of PS-latex was 0.009 mg/mL and light scattering was observed at a scattering angle of 90° in the figure. The filled circles in Figure 1a indicate the raw data, and the red line is the linear least-squares fit with the observed photon correlation function according to Equation 2.

A typical example of plots of apparent diameter *d_l,app_* calculated from the first cumulant of PS-latex against the PS-latex concentration and observed scattering angles is presented in Figure 1b. For the aqueous suspension of PS-latex, we performed 40 independent DLS measurements in the concentration range from 0.009 to 0.044 mg/mL and light scattering was observed at 8 scattering angles from 45° to 150°. In all the measurements, the observed raw photon correlation function showed linear decay similarly to the result in Figure 1a, but the slopes were different for corresponding angles and concentrations of PS-latex suspensions. There was almost a linear dependence of *d_l,app_* on concentration for each investigated concentration and**scattering angle.

**Figure 2:**
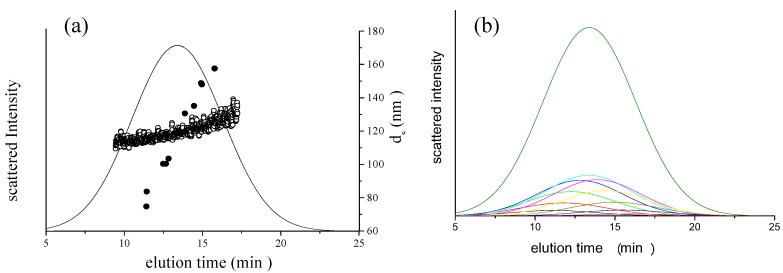
(**a**) Example of light scattering intensity fractogram (solid curves) and diameter of the PS-latex nanoparticles measured by AFFFF-MALS. The open circles denote the directly observed diameter determined by MALS. The filled circles denote the size of the PS-latex nanoparticles with narrow size distribution. The separation conditions are as follows: channel flow rate, 1.00 mL/min; cross flow rate, 0.25 mL/min. (**b**) Example of deconvolution of the fractogram in Figure 3a (black curves). The color curves illustrate the deconvoluted fractograms according to Equation 9.

The observed behavior in Figure 1b can be represented quantitatively by the following equation:


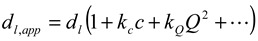
(8)

where *d_l_* is the true diameter, *k_c_* and *k_Q_* are constants, and *c* is the concentration of PS-latex nanoparticles in the suspension. The *d_l_*, *k_c_*, and *k_Q_* values for a specific PS-latex concentration and observed scattering angle in Equation 8 are determined by extrapolating the corresponding PS-latex concentration and observed scattering angle to zero in Figure 1b. Cichocki *et al.* [21] described the relationship between diffusion coefficients and interactions between nanoparticles by using the “effective radius,” which represents not only hydrodynamic interaction but also electrostatic repulsion and attraction at longer distances. Figure 1b and Table S-1 (see Supplementary Information) clearly show that the dynamics of PS-latex in aqueous suspension are dominated by long-range electrostatic interactions between PS-latex nanoparticles since *d_l,app_* shows clear dependence on angle and concentration. The particle scattering factor determined by static light scattering and the existence of electrostatic interactions between particles suggest a large angular distribution, as shown in Figure 1b and Table S-2 (see Supplementary Information). The effect of polydispersity of PS-latex nanoparticles and multiple scattering is not responsible for these dependences because (i) higher polydispersity makes the observed particle sizes larger at lower angles, and (ii) the most concentrated sample with the largest particle size did not exhibit multiple scattering effects at various intensities of the incident beam. Therefore, the double extrapolation of concentration and angular dependences in the DLS results can be used to determine *d_l_* in Equation 8. For example, in the Zimm plot analysis in Figure 2b, the *d_l_* value is estimated to be 118.2 nm. We carried out four independent measurements for Zimm plot analysis and found the average *d_l_* value was 118.5 nm.

**Figure 3:**
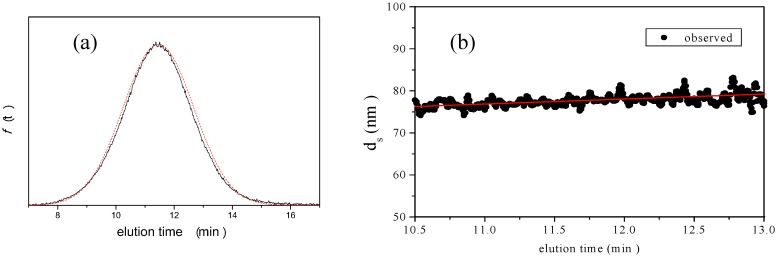
Example of AFFFF-MALS results for PS-latex (SC-0070-D). Channel flow rate, 1.00 mL/min; cross flow rate, 0.22 mL/min. (**a**) Apparent l ight scattering intensity ( black solid curve) fractogram and fitting curve according to Equation 12 (red solid curve). (**b**)   Apparent diameter of PS-latex (SC-0070-D) nanoparticles measured by MALS at corresponding elution times (filled black circles) and theoretical relationship between diameter and elution time (red line).

#### 3.1.3. Concept of Identifying and Analyzing Sources of Uncertainty in DLS

To estimate the sources of uncertainty, we use the GUM approach since the international guide for the estimation of the uncertainty was published by the standardization bodies. The individual sources of uncertainty in the intensity-average diameter (*d_l_*) of the PS-latex nanoparticles in an aqueous suspension were determined as shown in the Supplementary Information. The considered sources of uncertainty were the Boltzmann constant (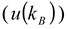) [22], the temperature (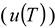), the solvent viscosity (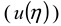) [20,23], the delay rate (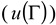), the solvent refractive index (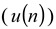) [24,25], the wavelength (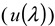), the observed scattering angle (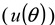), the repeatability of DLS measurements (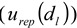), and the extrapolation to infinite dilution and to zero angle in the Zimm plot (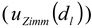).

The results of the uncertainty analysis are summarized in Table S-3 (see Supplementary Information). The combined uncertainty according to the GUM method is 0.69 nm.

### 3.2. AFFFF-MALS

#### 3.2.1. Calculation Method for Determining Size and Size Distribution by AFFFF-MALS

Figure 2a shows a fractogram obtained by AFFFF-MALS measurement of a PS-latex suspension (T0625) having a wide size distribution. The open circles indicate the apparent diameter determined by MALS for each separated fraction at 0.125 s intervals by AFFFF. The filled circles indicate the observed diameter corresponding to the peak in a fractogram for PS-latex nanoparticles that have a narrow size distribution (samples SC-0070-D, SC-0080-D, SC-100-D, SC-110-D, SC-0140-D, SC-016-S, F0223, T2112, T0622, T0021, T0118, and T0408). Although we do not have data on coefficient of variation (CV) values for the size distribution determined by DMA for samples F0223, T2112, T0622, T0021, T0118, and T0408, the almost flat nature of the plots of apparent diameters determined by the AFFFF-MALS indicates that their size distributions are moderately narrow in contrast to the plot of T0625 indicated by the open circles in Figure 2a. Although the plot of open circles shows a slope with respect to time, indicating that the T0625 has a wide size distribution, the open and filled circles are not fitted for the same elution times. This phenomenon indicates that the band broadening effect occurred in AFFFF separation. For AFFFF-MALS analysis of PS-latex nanoparticles, the accurate estimation of band broadening is important in both AFFFF separation and size distribution determination, although band broadening has little effect on the average size. Band broadening was therefore estimated as follows in this study.

To perform deconvolution of the fractogram, the obtained fractogram in Figure 2a was fitted to the following Gaussian function:


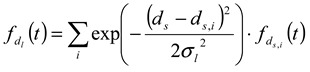
(9)

where


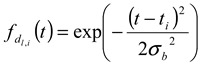
(10)

In this study, we employed a Gaussian function since the obtained AFFFF-MALS results are in good agreement with the function. In Equation 9, 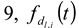 is a Gaussian function for truly monodisperse PS-latex nanoparticles assumed to have a standard deviation factor caused by band broadening (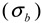) and to have a center of diameter from the set of positive integers. 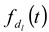 is composed of 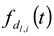  and 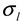 , which are in the set of positive integers. In 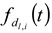  , 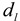  is the intensity-average diameter, and 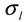 is the standard deviation of size distribution of PS-latex. Using these equations, the size distribution of PS-latex nanoparticles in an aqueous suspension is therefore assumed to be a Gaussian distribution and the fractogram can be represented as a function of the elution time for corresponding diameters of PS-latex nanoparticles. Under this assumption, the plot in Figure 2a changes to the one in Figure 2b.

This result indicates that, to accurately determine the nanoparticle size distribution, the plots of diameter directly observed by MALS (open circles in Figure 2a) cannot be used to form an appropriate calibration curve unless one considers the band broadening effect. In this study, therefore, we first determined 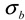  by using a PS-latex nanoparticle standard whose size distribution has already been well characterized by the DMA method [13]. Under this assumption, 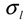   and 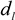  are then determined from the AFFFF-MALS results.

#### 3.2.2. Determination of Size and Size Distribution by AFFFF-MALS

**Figure 4:**
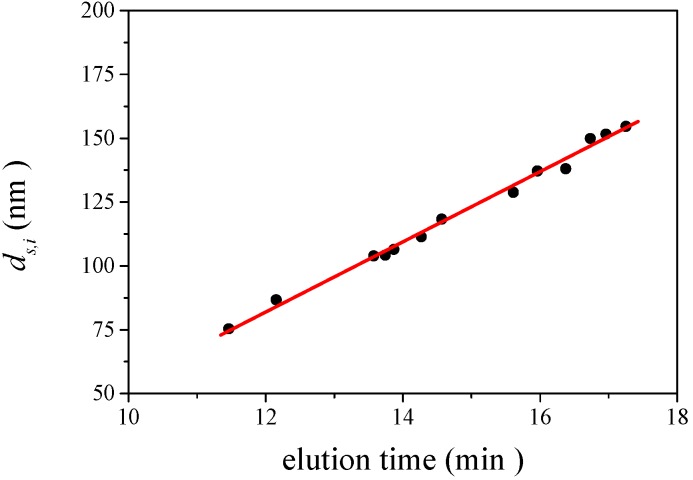
Example of observed relationship between separated size and elution time, as expressed by Equation 11.

Figure 3 shows the results of AFFFF-MALS for PS-latex (SC-0070-D). Figure 3a shows the apparent light scattering intensity fractogram, and Figure 3 b shows the apparent diameters of PS-latex nanoparticles measured by MALS at corresponding elution times. Under the same separation conditions (1.00 mL/min channel flow rate; 0.22 mL/min cross flow rate), the true relationship between nanoparticle size and elution time in the AFFFF system, as shown in Figure 4 by filled circles, is given by


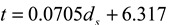
(11)

This linear relationship between separated size and elution time in Figure 4 was obtained from the AFFFF-MALS results for various PS-latex suspensions (SC-0070-D, SC-0080-D, SC-100-D, SC-110-D, SC-0140-D, SC-016-S, F0223, T2112, T0622, T0021, T0118, T0625, and T0408) . Substitution of Equation 10 into Equation 9 yields


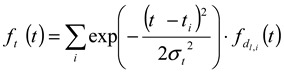
(12)

The standard deviation factor in Equation 10 is caused by band broadening in AFFFF separation (*σ _b_*), and by the standard deviation of the size distribution of PS-latex nanoparticles (*σ _l_*).

By using Equations 9 and 12, and changing the variable value (*σ _l_*), the AFFFF-MALS results for PS-latex (SC-0070-D) were theoretically fitted as shown by the red curve and line in Figures 3a and 3b, respectively. The PS-latex (SC-0070-D) has already been well characterized by DMA [18,26]. The standard deviation of the size distribution of the number-average diameter determined by DMA was 5.1 nm, and therefore the standard deviation of the size distribution of the calculated intensity-average diameter assuming a Gaussian size distribution was 4.9 nm. From Equation 10, the appropriate *σ _b_* value for the experimental data was calculated to be 1.17 min. To examine the correctness of this method for estimating *σ _l_* values, we calculated the CV values for five independent PS-latex suspensions in Table 2, respectively. The official CV values are calculated from the standard deviation of the size distribution determined by DMA, and the observed size distributions are grouped by size. The calculated CV values for these PS-latex suspensions were estimated from the standard deviation of the size distribution by AFFFF-MALS. The calculated CV values agreed well with the respective experimental values, indicating that *σ _b_* calculated through this procedure was appropriate.

**Table 2:** Summary of calculated CV values for various PS-latex nanoparticles by AFFFF-MALS.

Sample name	Official CV value	Calculated CV value
%	%
STADEX SC-0080-D	4.80	4.60
STADEX SC-0100-D	2.47	2.20
STADEX SC-0110-D	3.10	3.30
STADEX SC-0140-D	1.42	1.30
STADEX SC-016-S	2.46	2.20

**Figure 5:**
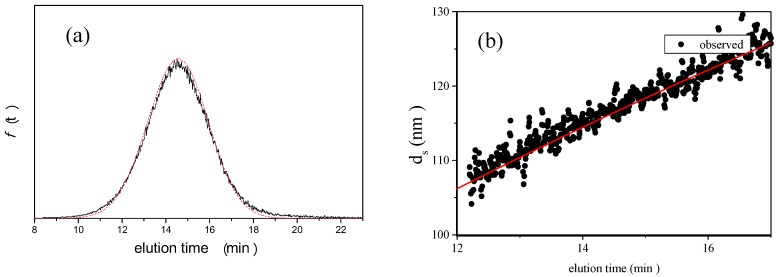
AFFFF-MALS results for **PS-latex ** (T0625). **Channel flow rate, 1.00 mL/min; cross flow rate, 0.22 mL/min. ** The calculated *d_l_* is 117.2 and 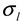 is 11.5 nm from Equation 15. (a) Apparent l **ight scattering intensity ( black solid curve) fractogram and fitting curve expressed by Equation 12 (red solid curve). ( ** b**)Apparent diameter of PS-latex ** (T0625) **nanoparticles measured by MALS at corresponding elution times (filled black circles) and theoretical relationship between diameter and elution time (red line) . **

Figure 5 shows the results of AFFFF-MALS for PS-latex (T0625). Figure 5a shows the apparent light scattering intensity fractogram, and Figure 5 b shows the apparent diameter of PS-latex nanoparticles measured by MALS at corresponding elution times. In Figure 5, by using Equation 9 and 12 and by changing the variable value (*σ _l_*) to have a fixed *σ _b_* value of 1.17 min, the AFFFF-MALS results for PS-latex (T0625) agreed well with theory as shown by the red curve (Figure 5a) and the red line (Figure 5b) in the figures. In Figure 5, the calculated *d_l_* is 117.2 and *σ _l_* is 11.5 nm from Equations 9 and 12. We carried out 12 independent measurements (3 measurements performed under 4 different cross flow rate conditions: 0.18, 0.20, 0.22, and 0.25 mL/min) and the average *d_l_* and *σ _l_* were found to be 117.6 and 10.1 nm, respectively.

#### 3.2.3. Concept of Identifying and Analyzing Sources of Uncertainty in AFFFF-MALS

We also identified the sources of uncertainty in AFFFF-MALS as well as those in DLS. Individual sources of uncertainty are described in the Supplementary Information. We considered the sources of uncertainty from the theoretical fitting for light scattering in MALS measurement (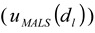), the procedure for determining the baseline for AFFFF-MALS measurement (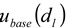 and 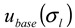), the procedure for determining the band broadening factor (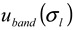), the calibration line in Equation 11 (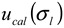), and the repeatability of the AFFFF-MALS measurements (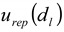 and 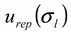). The results of separate uncertainty analyses are summarized in Tables S-4 and S-5 (see Supplementary Information). The combined standard uncertainty values calculated for *d_l_* and 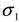 for PS-latex (T0625) are 3.48 and 0.38 nm, respectively.

### 3.3. Comparison of DLS and AFFFF-MALS Results

Having accurately determined the particle size, size distribution, and uncertainties for both DLS and AFFFF-MALS measurements, we next compared the results of DLS and AFFFF-MALS; the uncertainties are necessary to compare the results obtained from different metrological methods. To compare the determined diameters by these two methods, the intensity-average diameter was employed in this study, since the raw particle size determined by the DLS method is the intensity-average diameter. The weight- or number-average diameters of PS-latex nanoparticles determined by DLS are calculated from the average results from light scattering intensity under simple assumptions (size distribution, spherical structure, *etc.*). There are many commercial instruments and analytical methods that are based on various DLS principles; however, it is well known that, as determined by DLS methods, the weight- and number-average diameter of nanoparticles and the calculated size distribution strongly depend on instrument and analytical procedure [13]. We therefore used the intensity-average diameter for comparing the diameters determined by DLS and AFFFF-MALS.

**Figure 6:**
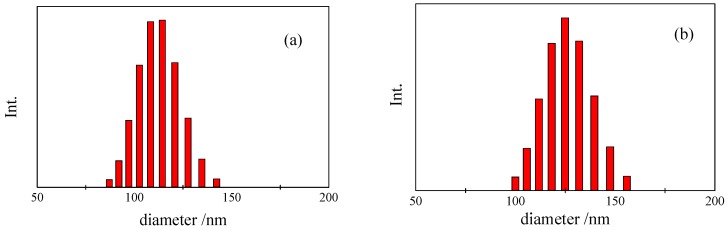
Examples of size distribution for PS-latex nanoparticle suspensions determined by DLS using cumulant analytical method. (**a**) STADEX SC-0110-D and (**b**) T0625.

**Figure 7:**
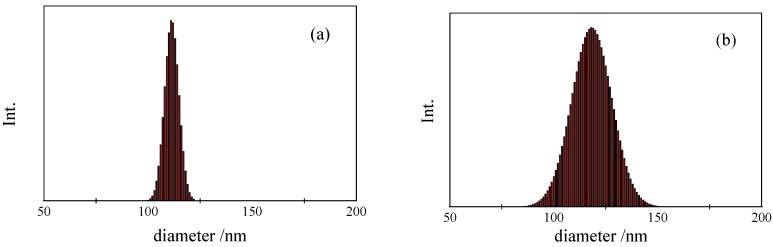
Examples of size distribution for PS-latex nanoparticle dispersions determined by AFFFF-MALS . (**a**)STADEX SC-0110-D and (**b**) T0625.

Figure 6 shows the size distribution of PS-latex nanoparticle suspensions (STADEX SC-0110-D and T0625) determined by DLS by cumulant analysis and by AFFFF-MALS. The size distributions of the two PS-latex nanoparticles determined by DLS were found to be similar (Figure 6a and 6b). However, the size distributions of the same two PS-latex nanoparticles determined by AFFFF-MALS were found to be markedly different (Figure 7a and 7b), with T0625 nanoparticles showing a wider size distribution. Interestingly, the average diameter of PS-latex nanoparticles at the peak of the size distribution histograms in Figures 6 and 7, determined by both DLS and AFFFF-MALS are almost the same. These results indicate that the size distribution determined by DLS is not reliable in contrast to the intensity-average diameter. This implies that the weight- and number-average diameters obtained by DLS are unreliable, since those values calculated from the intensity-average diameter by DLS are strongly dependent on the light scattering intensity size distribution.

Focusing on the results for T0625, the intensity-average diameter determined by DLS is 118.5 ± 0.69 nm. On the other hand, the value determined by AFFFF-MALS is 117.7 ± 3.48 nm, indicating that the particle size determined by dynamic and static light scattering methods (DLS and AFFFF-MALS) agree well within the estimated uncertainties when using the accurate analytical method established in this study. The values determined by DLS are slightly larger than those determined by AFFFF-MALS. This result might be attributable to the existence of an electrical double layer around the PS-latex particles on account of strongly adsorbed water [27]. In a previous investigation, the thickness of the adsorbed water monolayer on sodium dodecyl sulfate micelles was estimated to be 0.51 nm [28]. Although several methods based on AFFFF-MALS, TEM, SEM, and DLS deal with comparisons of measured average sizes [29,30,31,32], our method is notable in that a precise DLS and AFFFF-MALS analytical protocol and careful uncertainty analysis are used, which brings out the best in the two metrologies since previous comparisons used a simple linear curve fitting of MALS measurements without taking into account band broadening to estimate the size distribution in AFFFF-MALS. This study, to the best of our knowledge, is therefore the first attempt to compare sizes determined by these two different methods. In addition, the methods developed here could be a useful general protocol for determining size and size distribution in a liquid phase and could be of great benefit to practitioners.

## 4. Conclusions

In this study, we investigated a practical protocol for evaluating the size and size distribution of PS-latex nanoparticles in an aqueous suspension by using DLS and AFFFF-MALS analyses. First, we established a novel and practical protocol for accurately determining particle size and size distribution, considering various sources of uncertainty according to the GUM method. The intensity-average diameters determined by DLS and AFFFF-MALS agreed well within the estimated uncertainty, indicating that the particle sizes determined by the dynamic and static light scattering methods were well within the estimated uncertainty when using our analytical protocol. However, the size distribution of PS-latex nanoparticles determined by DLS was less reliable in comparison to the values obtained by AFFFF-MALS. This novel protocol could be a practical method for researchers to achieve some degree of concordance in nanoparticle size determination in industrial and biological research.

## Supplementary Materials

Supplementary File 1
