# Supplementary material for: Accurate Size and Size-Distribution Determination of Polystyrene Latex Nanoparticles in Aqueous Medium Using Dynamic Light Scattering and Asymmetrical Flow Field Flow Fractionation with Multi-Angle Light Scattering
Source: Nanomaterials (Basel). 2012 Jan 5;2(1):15–30. doi: 10.3390/nano2010015 (PMC5327882; doi:10.3390/nano2010015)
Supplement: Supplementary File 1 [file nanomaterials-02-00015-s001.pdf]

Nanomaterials **2012**, *2*, 15-30; doi:10.3390/nano2010015

OPEN ACCESS

nanomaterials

ISSN 2079-4991

www.mdpi.com/journal/nanomaterials

Article

Accurate Size and Size-Distribution Determination of Polystyrene Latex Nanoparticles in Aqueous Medium Using Dynamic Light Scattering and Asymmetrical Flow Field-Flow Fractionation with Multi-Angle Light Scattering

Haruhisa Kato *, Ayako Nakamura, Kayori Takahashi and Shinichi Kinugasa

Polymer Standards Section Japan (PSSJ), Particle Measurement Section (PMS), National Metrology Institute of Japan (NMIJ), National Institute of Advanced Industrial Science and Technology (AIST), Tsukuba Central 5, 1-1-1 Higashi, Tsukuba, Ibaraki 305-8565, Japan;
E-Mails: nakamura@tasc-nt.or.jp (A.N.); kayori.takahashi@ni.aist.go.jp (K.T.);
s.kinugasa@aist.go.jp (S.K.)

* Author to whom correspondence should be addressed; E-Mail: H-kato@aist.go.jp;
Tel.: +81-29-861-489; Fax: +81-29-861-4618.

Supplementary Information

1. Uncertainty Sources in DLS

1.1. Boltzmann Constant (k_B)

The Boltzmann constant is 1.3806504 × 10^{-23} J/K and the standard uncertainly is 0.000000024 × 10^{-23} J/K according to the literature [17].

1.2. Temperature (T)

The estimated value of temperature variation in DLS, measured by ISOTECH TTI-7 thermometer, was 0.1 *K*. The standard deviation of temperature was calculated using the assumption of a rectangular distribution for that variation, *i.e.*, $0.1\ K/\sqrt{3} = 0.0577\ K$.

1.3. Viscosity (η)

The viscosity of the solvent used in this study was 0.8902 cP [15]. According to the literature [18], the relative standard uncertainly is 0.01%.

1.4. Delay Rate (Γ)

The diffusion coefficient of PS-latex is calculated by Equation 3. Using the linear least-squares fitting procedures, one can first calculate Γ as the slope of the fitting line of the plot of experimental values of ln $(g_1(\tau))$ as a function of τ. The uncertainty of Γ arises from the deviation of the experimental data points from the linear least-squares line. The uncertainty of Γ calculated by Equations S-1 and S-2 is 5.0.

$$u(\Gamma) = \frac{s_{y/x}(\Gamma)}{\left\{\sum_i \left(\tau^2 - \overline{\tau^2}\right)^2\right\}^{0.5}} \quad \text{(S-1)}$$

where,

$$s_{y/x}(\Gamma) = \left\{\frac{\sum_{i=1} [\ln(g_1(\tau)_i) - \ln(\hat{g}_1(\tau)_i)]^2}{n-2}\right\}^{0.5} \quad \text{(S-2)}$$

*1.5. Refractive Index of Water (*n*)*

The value of refractive index of water used in this study was 1.33230 mL/g [19]. Other literature [20] reported refractive index of water as 1.33171 mL/g. We therefore assumed a rectangular distribution of the difference between these values and calculated the uncertainly as $u(n) = 0.00059$ mL/g.

1.6. Wavelength (λ)

In this study, we used He-Ne laser at a wavelength of 632.8 nm. The estimated value of wavelength variation was 0.001 nm. The standard deviation of wavelengthwas calculated using the assumption of a rectangular distribution for that variation, *i.e.*, $0.001\, nm/\sqrt{3} = 0.000577\, nm$.

1.7. Scattering Angle (θ)

The estimated value of variation of observed scattering angle in DLS was found to be 0.1 degree. The standard deviation of the observed scatteringangle was calculated using the assumption of a rectangular distribution for that variation, *i.e.*, $0.000617\, rad/\sqrt{3} = 0.000356\, rad$.

1.8. Repeatability of DLS Measurements

The standard uncertainties of the size of sondary nanoparticles, $u_{\text{rep}}(d_1)$, arising from repeated DLS measurements coupled with a change in the size of the particles is given by:

$$u_{rep}(d_l) = \sqrt{\frac{\sum_{i=1}^{n} (d_{l,i} - d_l)^2}{n(n-1)}} \quad \text{(S-3)}$$

where, n is the number of measurements. The estimated uncertainty from four independent measurements is 0.68 nm.

1.9. Extrapolation to Infinite Dilution and to Zero Angles (Zimm Plot)

Using the linear least-squares fitting procedure, one can first estimate the true hydrodynamic diameter as the intercept of the fitting line of the plot of experimental values of c as a function of angle. The uncertainty of the extrapolation to infinite dilution and to zero angles arises from the deviation of the experimental data points from the linear least-squares line.

$$u_{Zimm}(d_l) = s_{y/x}(d_l)\left\{\frac{\sum_i Q_i^2}{n\sum_i (Q_i - \bar{Q})^2}\right\}^{0.5} \quad \text{(S-4)}$$

where,

$$s_{y/x}(d_l) = \left\{\frac{\sum_{i=1}\left[d_i - \hat{d}_i\right]^2}{n-2}\right\}^{0.5} \quad \text{(S-5)}$$

The uncertainty calculated by Equations (S-4) and (S-5) is 0.1 nm.

1.10. Calculation of the Combined Standard Uncertainty of Light Scattering Intensity Averaged Diameter by DLS

The **c**alculation of the combined standard uncertainty of light scattering intensity averaged diameter by DLS was performed as follows.

According to Stokes-Einstein assumption (Equation 5), the light scattering intensity averaged diameter determined by DLS was calculated by:

$$d_l = \frac{k_B T Q^2}{3\pi\eta\Gamma} = \frac{16\pi k_B T n^2}{3\eta\lambda^2\Gamma}\sin^2\left(\frac{\theta}{2}\right) \quad \text{(S-6)}$$

The standard uncertainty of d_l is calculated by

$$u^2(d_l) = \left(\frac{\partial d_l}{\partial k_B}\right)^2 u^2(k_B) + \left(\frac{\partial d_l}{\partial T}\right)^2 u^2(T) + \left(\frac{\partial d_l}{\partial \eta}\right)^2 u^2(\eta) + \left(\frac{\partial d_l}{\partial \Gamma}\right)^2 u^2(\Gamma) + \left(\frac{\partial d_l}{\partial n}\right)^2 u^2(n) + \left(\frac{\partial d_l}{\partial \lambda}\right)^2 u^2(\lambda) + \left(\frac{\partial d_l}{\partial \theta}\right)^2 u^2(\theta) + u_{rep}^2(d_l) + u_{Zimm}^2(d_l) + u_{\mathrm{homo}}^2(d_l) \quad \text{(S-7)}$$

which, when expanded becomes:

$$u^2(d_l)=\left(\frac{16\pi Tn^2}{3\eta\lambda^2\Gamma}\sin^2\left(\frac{\theta}{2}\right)\right)^2 u^2(k_B)+\left(\frac{16\pi k_B n^2}{3\eta\lambda^2\Gamma}\sin^2\left(\frac{\theta}{2}\right)\right)^2 u^2(T)$$
$$+\left(-\frac{16\pi k_B Tn^2}{3\eta^2\lambda^2\Gamma}\sin^2\left(\frac{\theta}{2}\right)\right)^2 u^2(\eta)+\left(-\frac{16\pi k_B Tn^2}{3\eta\lambda^2\Gamma^2}\sin^2\left(\frac{\theta}{2}\right)\right)^2 u^2(\Gamma)+\left(\frac{32\pi k_B Tn}{3\eta\lambda^2\Gamma}\sin^2\left(\frac{\theta}{2}\right)\right)^2 u^2(n) \quad \text{(S-8)}$$
$$+\left(-\frac{32\pi k_B Tn^2}{3\eta\lambda^3\Gamma}\sin^2\left(\frac{\theta}{2}\right)\right)^2 u^2(\lambda)+\left(\frac{8\pi k_B Tn^2}{3\eta\lambda^2\Gamma}\sin(\theta)\right)^2 u^2(\theta)+u_{rep}{}^2(d_l)+u_{Zimm}{}^2(d_l)$$

2. Uncertainty Sources in AFFFF-MALS

2.1. Fitting for Light Scattering in MALS Measurement

Due to use of Equation 7 in the theoretical fitting procedure of apparent light scattering intensity profiles in the corresponding angles in MALS measurement, it is difficult to estimate the standard uncertainty from the procedure analytically. Therefore, the standard uncertainties in the fitting procedure of light scattering intensity profiles, $u_{\mathrm{MALS}}(d_1)$, were estimated by:

$$\frac{u_{MALS}^2(d_l)}{d_l^{\,2}}=\frac{\Delta_{d_l}{}^2}{3d_l^{\,2}}=\frac{(d_{l,\max}-d_{l,\min})^2}{12d_l^{\,2}} \quad \text{(S-9)}$$

with a change in the size of the particles in Equation 7. The maximum diameter size ($d_{l,\max}$) and minimum diameter size ($d_{l,\min}$) that has a good agreement between the experimental data and the simulated data were estimated. Then, the $u_{\mathrm{MALS}}(d_1)$ was calculated according to Equation (S-9).

2.2. Baseline for AFFFF-MALS Measurement

It is difficult to estimate the standard uncertainty from the procedure to determine the base line analytically. We therefore changed the way of drawing the base line in AFFFF chromatogram and evaluated the maximum diameter size ($d_{\max}$) and minimum diameter size ($d_{\min}$) that agree as correct baseline for the experimental data as shown. The standard uncertainties from the drawing procedure for baseline determination in AFFFF measurements for calculation of $u_{\mathrm{base}}(d_1)$and $u_{\mathrm{base}}(\sigma_1)$ are as follows:

$$\frac{u_{base}^2(d_l)}{d_l^{\,2}}=\frac{\Delta_{d_l}{}^2}{3d_l^{\,2}}=\frac{(d_{l,\max}-d_{l,\min})^2}{12d_l^{\,2}} \quad \text{(S-10)}$$

$$\frac{u_{base}^2(\sigma_l)}{\sigma_l^{\,2}}=\frac{\Delta_{\sigma_l}{}^2}{3\sigma_l^{\,2}}=\frac{(\sigma_{l,\max}-\sigma_{l,\min})^2}{12\sigma_l^{\,2}} \quad \text{(S-11)}$$

2.3. Band Broadening

It is difficult to estimate the standard uncertainty from the procedure to give the band broadening factor (σ_b) using Equation 15 analytically. We therefore changed the fitting procedure in AFFFF-MALS analysis and evaluated the maximum ($\sigma_{b,\max}$) and minimum band broadening factor

($\sigma_{b,\min}$) that agrees as fitting curve and line for the experimental data as shown in Figure 6. The standard uncertainties from the band broadening factor used in determination of the standard deviation of the size distribution, $u_{\text{band}}(\sigma_l)$, is calculated by the following equation:

$$\frac{u_{band}^2(\sigma_l)}{\sigma_l^{\ 2}} = \frac{\Delta_{\sigma_l}^{\ 2}}{3\sigma_l^{\ 2}} = \frac{(\sigma_{l,\max} - \sigma_{l,\min})^2}{12\sigma_l^{\ 2}} \tag{S-12}$$

For calculating the value of $u_{band}(\sigma_l)$, the appropriate range of the band broadening factor is 1.15–1.19 nm, and the values of $\sigma_{l,\max} - \sigma_{l,\min}$ using $\sigma_{b,\max}$ and $\sigma_{b,\min}$ is 0.4 nm.

2.4. Calibration Line

For determination of the calibration line in Equation 14, for example, by the linear least square fitting, the uncertainty of the d_l was calculated by the following equations:

$$u_{cal}(d_l) = s_{y/x}(d_l)\left\{1 + \frac{1}{n} + \frac{\left(d_{l,i} - \overline{d_{l,i}}\right)^2}{b^2 \sum_i \left(t_i - \overline{t_i}\right)^2}\right\}^{0.5} \tag{S-13}$$

where,

$$s_{y/x}(d_l) = \left\{\frac{\sum_{i=1} \left[d_{l,i} - \hat{d}_{l,i}\right]^2}{n-2}\right\}^{0.5} \tag{S-14}$$

In this study, the calculated uncertainty is 3.46 nm.

2.5. Repeatability of AFFFF-MALS Measurements

The standard uncertainties of the size of sondary nanoparticles and the standard deviation of the size distribution, $u_{\text{rep}}(d_1)$ and $u_{\text{rep}}(\sigma_1)$, respectively, arising from repeated independent AFFFF-MALS measurements coupled with a change in size of the particles is given by:

$$u_{rep}(d_l) = \sqrt{\frac{\sum_{i=1}^{n} \left(d_{l,i} - d_l\right)^2}{n(n-1)}} \tag{S-15}$$

$$u_{rep}(\sigma_l) = \sqrt{\frac{\sum_{i=1}^{n} \left(\sigma_{l,i} - \sigma_l\right)^2}{n(n-1)}} \tag{S-16}$$

where, n is the number of measurements. The estimated uncertainties from 12 independent measurements (three measurements with corresponding different four cross flow rate conditions: 0.18, 0.20, 0.22, and 0.25 mL/min) for $u_{\text{rep}}(d_1)$ and $u_{\text{rep}}(\sigma_1)$ are 0.30 and 0.36 nm, respectively.

2.6. Calculation of the Combined Standard Uncertainty of Light Scattering Intensity Averaged Diameter and Size Distribution by AFFFF-MALS

The calculation of the combined standard uncertainties of light scattering intensity averaged diameter and size distribution by AFFFF-MALS ($u_{AFFFF-MALS}(d_l)$ and $u_{AFFFF-MALS}(\sigma_l)$) were calculated as follows. The standard uncertainty of d_l could be calculated by:

$$u_{AFFFF-MALS}{}^2(d_l) = u_{MALS}{}^2(d_l) + u_{base}{}^2(d_l) + u_{cal}{}^2(d_l) + u_{rep}{}^2(d_l) \quad \text{(S-17)}$$

$$u_{FFFF-MALS}{}^2(\sigma_l) = u_{base}{}^2(\sigma_l) + u_{band}{}^2(\sigma_l) + u_{rep}{}^2(\sigma_l) \quad \text{(S-18)}$$

Table S-1. Calculation results of d_l and k_c values for corresponding PS-latex (T0625) concentration in Equation 8.

**Concentration (mg/mL)**	**0.009**	**0.018**	**0.027**	**0.035**	**0.044**
d_l (nm)	117.4	117.1	116.2	115.6	115.0
k_c (mL/mg)	1.0×10^{-14}	0.8×10^{-14}	1.0×10^{-14}	0.9×10^{-14}	0.8×10^{-14}

Table S-2. Calculation results of d_l and k_Q values for corresponding observed scattering angles in Equation 8.

**Angle (degree)**	**45**	**60**	**75**	**90**
d_l (nm)	119.1	120.4	121.7	121.3
k_Q (nm^2)	−92.9	−95.3	−97.3	−92.2
**Angle (degree)**	**105**	**120**	**135**	**150**
d_l (nm)	122.9	124.7	125.1	125.0
k_Q (nm^2)	−107.0	−106.7	−106.9	−100.2

Table S-3. Summary of calculated uncertainty for PS-latex (T0625) by DLS.

**x**	**x values**	**u(x)**	**u(x)/x**	$\left(\frac{\partial f}{\partial x}\right)\frac{u(x)}{d_l}$
k_B	1.380658×10^{-23} m^2 kg s^{-2} K^{-1}	2.4×10^{-31} m^2 kg s^{-2} K^{-1}	1.7×10^{-8}	1.0×10^{-9}
T	298.15 K	5.77×10^{-2} K	1.9×10^{-4}	2.0×10^{-5}
η	8.9020×10^{-3} kg m s^{-1}	8.902×10^{-8} kg m s^{-1}	1.0×10^{-5}	1.0×10^{-6}
Γ	1414.02 s	5.0 s	3.5×10^{-3}	3.6×10^{-4}
n	1.3323	5.9×10^{-4}	4.4×10^{-4}	6.8×10^{-5}
λ	632.991×10^{-9} m	5.77×10^{-13} m	9.1×10^{-7}	1.9×10^{-7}
θ	1.5708 rad	3.57×10^{-4} rad	2.3×10^{-4}	1.2×10^{-5}
rep	118.5×10^{-9} m	6.8×10^{-10} m	5.7×10^{-3}	5.7×10^{-3}
Zimm	118.5×10^{-9} m	1.0×10^{-10} m	8.5×10^{-4}	8.5×10^{-4}
d_l	118.5 nm	6.9×10^{-1} nm	7.7×10^{-3}	7.7×10^{-3}

Table S-4. Summary of calculated uncertainty of d_l for PS-latex (T0625) by AFFFF-MALS.

**x**	**x values**	**u(x)**	**u(x)/x**	$\left(\frac{\partial f}{\partial x}\right)\frac{u(x)}{d_l}$
MALS	117.6 nm	0.23 nm	2.0×10^{-3}	2.0×10^{-3}
base	117.6 nm	0.06 nm	5.1×10^{-4}	5.1×10^{-4}
cal	117.6 nm	3.46 nm	2.9×10^{-2}	2.9×10^{-2}
rep	117.6 nm	0.30 nm	2.6×10^{-3}	2.6×10^{-3}
d_l	117.6 nm	3.48 nm	3.0×10^{-2}	3.0×10^{-2}

Table S-5. Summary of calculated uncertainty of σ_l for PS-latex (T0625) by AFFFF-MALS.

**x**	**x values**	**u(x)**	**u(x)/x**	$\left(\frac{\partial f}{\partial x}\right)\frac{u(x)}{\sigma_l}$
base	11.9 nm	0.06 nm	5.1×10^{-3}	2.0×10^{-3}
band	11.9 nm	0.12 nm	1.0×10^{-2}	5.1×10^{-4}
rep	11.9 nm	0.36 nm	3.0×10^{-2}	3.0×10^{-2}
σ_l	11.9 nm	0.38 nm	3.2×10^{-2}	3.2×10^{-2}

© 2012 by the authors; licensee MDPI, Basel, Switzerland. This article is an open access article distributed under the terms and conditions of the Creative Commons Attribution license (http://creativecommons.org/licenses/by/3.0/).
